# Supplementary material for: TDP-43 facilitates milk lipid secretion by post-transcriptional regulation of Btn1a1 and Xdh
Source: Nat Commun. 2020 Jan 17;11:341. doi: 10.1038/s41467-019-14183-1 (PMC6969145; doi:10.1038/s41467-019-14183-1)
Supplement: Supplementary file 1 — Supplementary Information [file 41467_2019_14183_MOESM1_ESM.pdf]

Supplementary Information

TDP-43 facilitates milk lipid secretion by post-transcriptional regulation of *Btn1a1* and *Xdh*

Zhao et al.,

Supplementary Figure 1-12

Supplementary Table 1-3

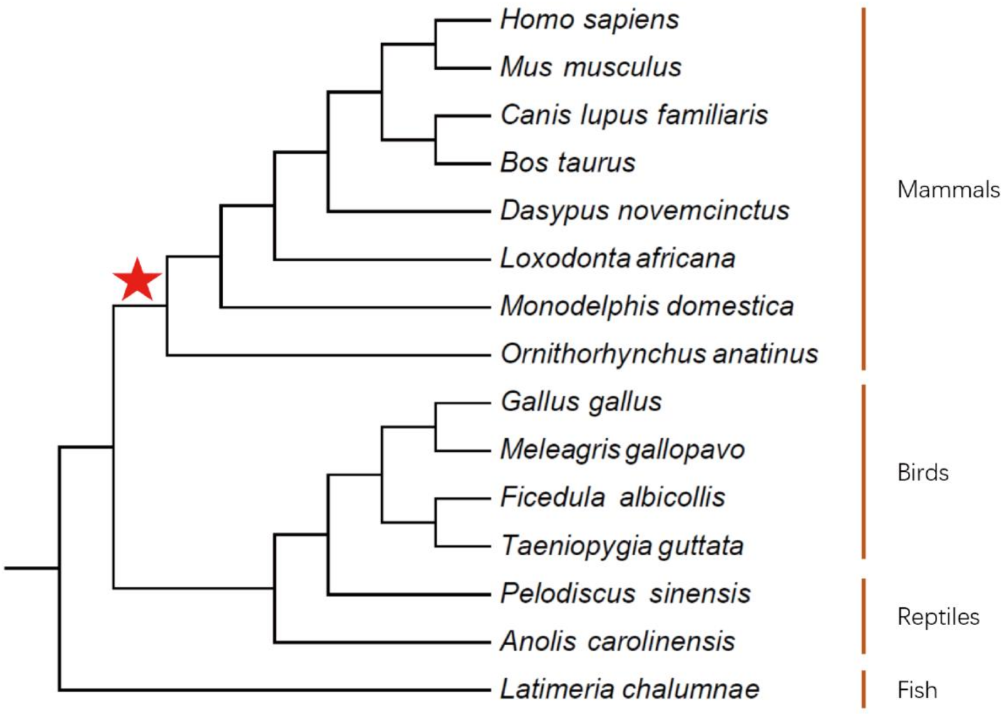

Supplementary Fig.1. Topology of 15 species used to estimate dN/dS and LRTs

Red star marks the branch of the most recent common ancestor of mammals.

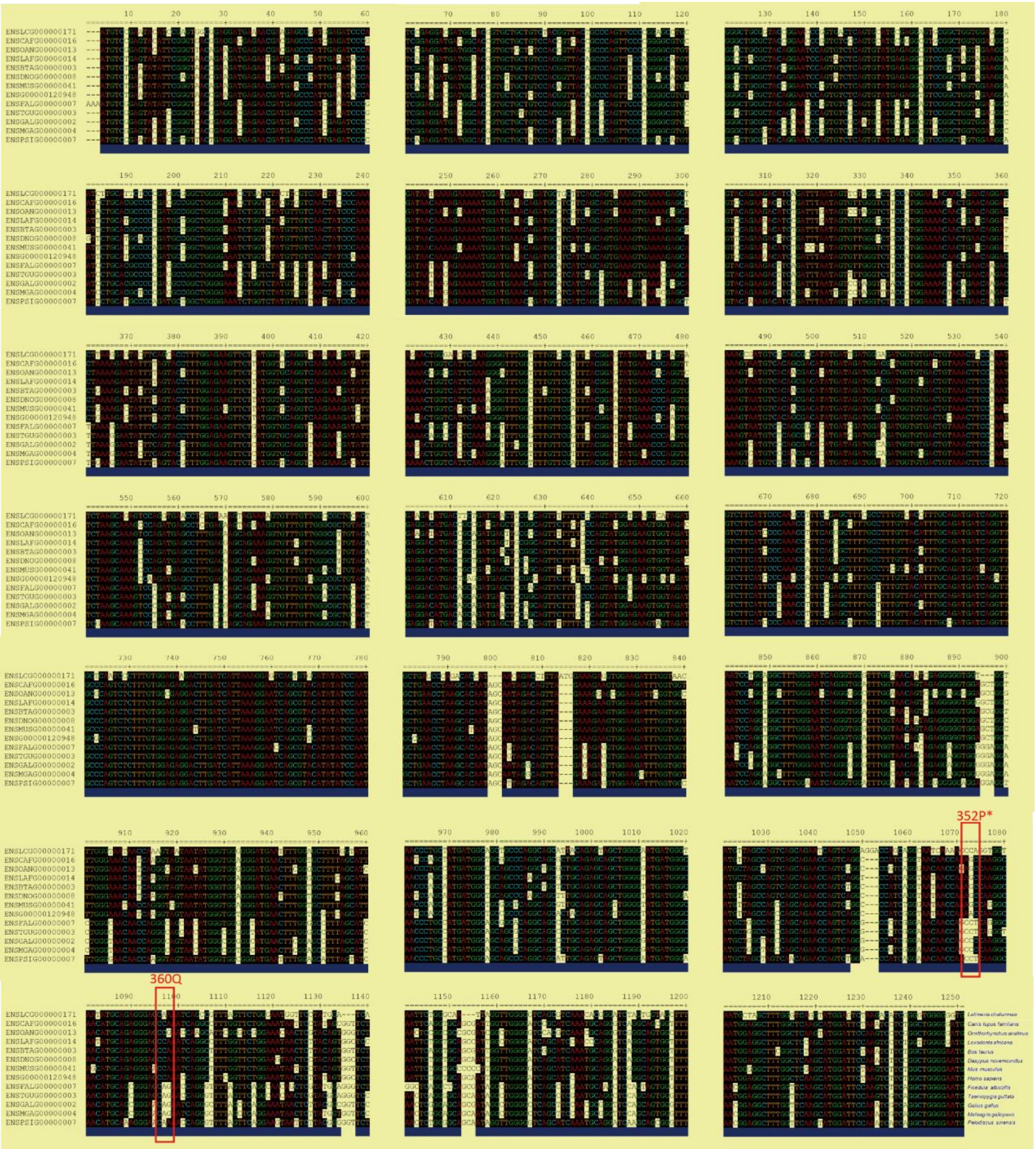

Supplementary Fig. 2. Multiple alignments and conserved codons of *TARDBP*

Multiple alignments contained a total of 1 251 nt. Conserved codons (1 227 nt) used to estimate dN/dS and LRTs are underlined in blue. Red rectangles indicate the positive selected sites. The numbers and letters above the red rectangles show the position and the type of positive selected amino acid residues (Table 1). * presents 5% significant level.

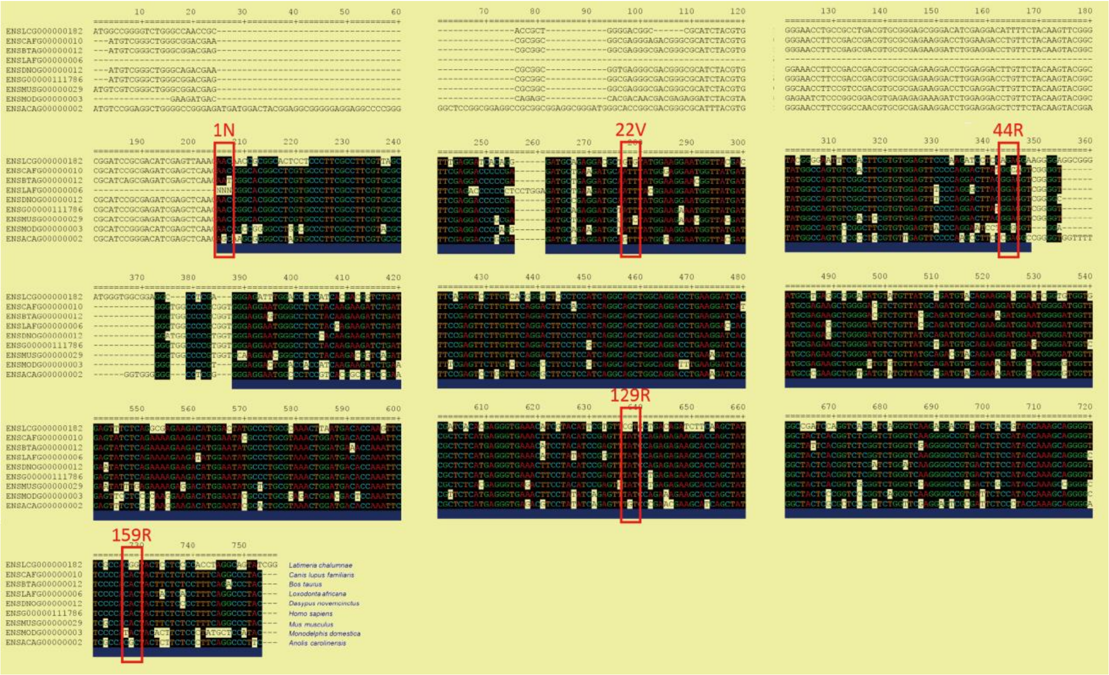

Supplementary Fig. 3. Multiple alignments and conserved codons of *SRSF9*

Multiple alignments contained a total of 756 nt. Conserved codons (504 nt) used to estimate dN/dS and LRTs are underlined in blue. Red rectangles indicate the positive selected sites. The numbers and letters above the red rectangles show the position and the type of positive selected amino acid residues (Table 1).

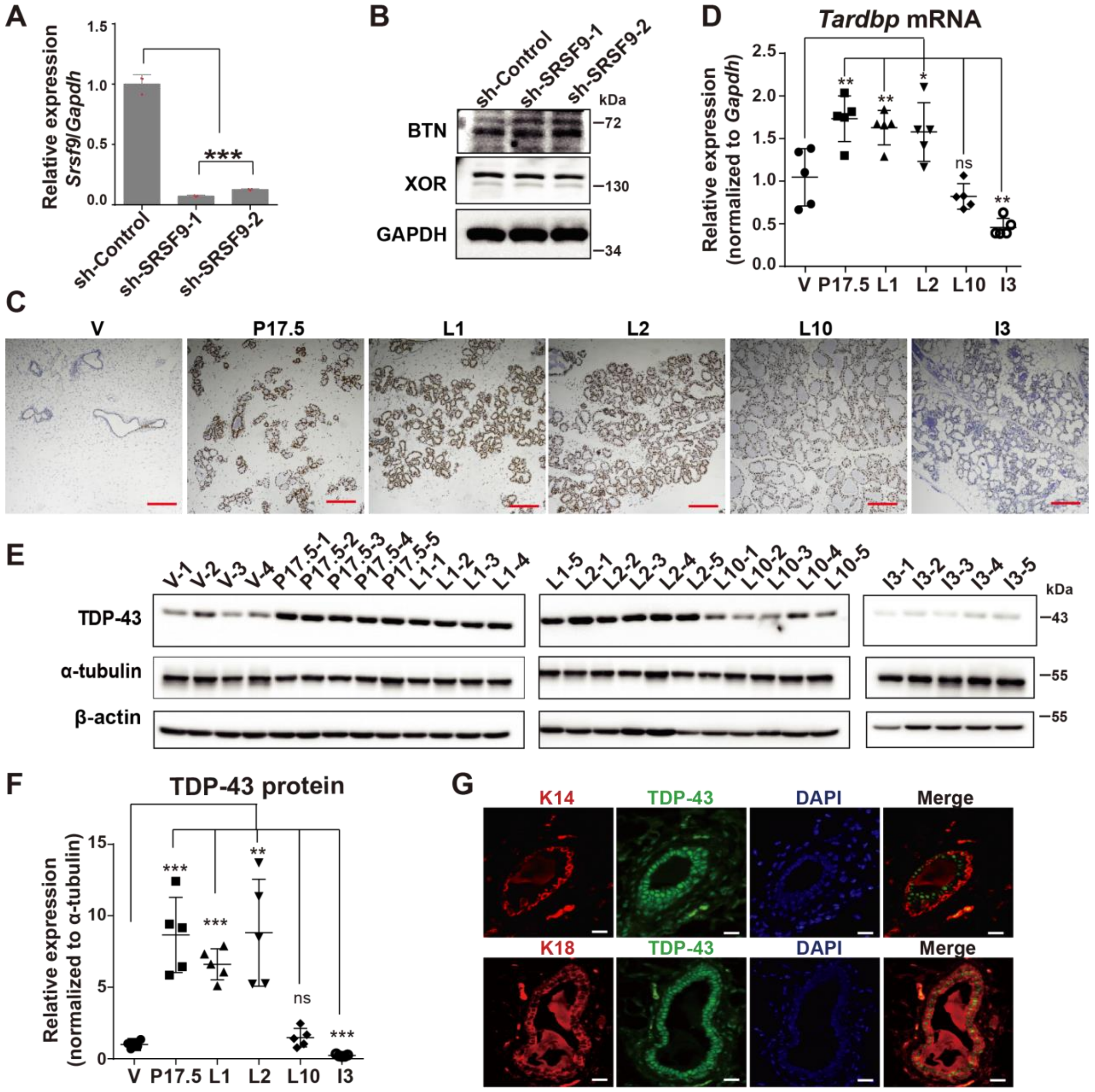

Supplementary Fig. 4. TDP-43 expression during mammary gland development

(A-B) Relative mRNA expression (A) and protein levels (B) of indicated genes upon sh-SRSF9 knockdown. Data were shown as the means ± SD of three independent experiments (A). (C) Immunohistochemical (IHC) staining of TDP-43 in mammary glands of C57BL/6 female mice at indicated developmental stages. Representative images (10× objective magnification) from four mice are shown for each stage. Scale bar: 200 μm. (D) *Tardbp* mRNA levels at indicated developmental stages from five mice in each group. (E and F) Western blot (E) and relative quantification (F) for TDP-43 protein levels at indicated developmental stages (four or five replicates for

each stage). Same amount of protein (40 μg/lane) was loaded in each Western blot lane. (G) Immunofluorescence staining of mammary glands for TDP-43 (green), K14 (red, upper), and K18 (red, bottom) in virgin mice (8 weeks). V, virgin; P17.5, pregnancy day 17.5; L1, lactation day 1; L2, lactation day 2; L10, lactation day 10; I3, involution day 3. Scale bar: 20 μm. Data are means ± SD. Unpaired *t*-test was used to evaluate statistical significance. ∗ P < 0.05, ∗∗ P < 0.01, ∗∗∗ P < 0.001. ns, not significant. Source data are provided as a Source Data file.

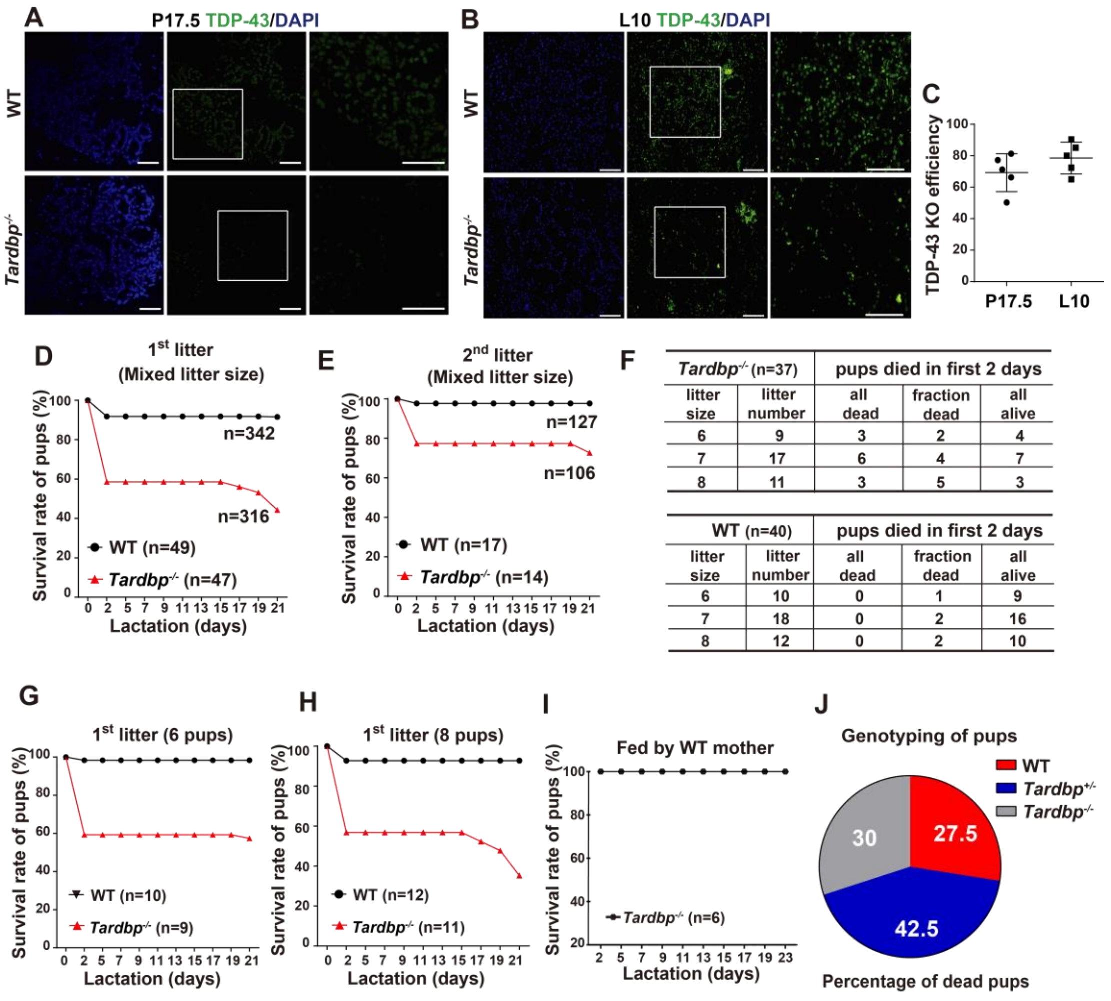

Supplementary Fig. 5. Conditional knockout of *Tardbp* in mammary glands

(A and B) Immunofluorescence staining to verify knockout efficiency of *Tardbp*$^{-/-}$ mice at pregnancy day 17.5 (P17.5) (A, scale bar: 50 μm) and lactation day 10 (L10) (B, scale bar: 200 μm), and statistical analysis of knockout efficiency (C). The results were from n=5 biologically independent sample. Data are means ± SD. (D and E) Overall pup survival rates in different litter sizes in first (D) and second (E) lactation. (F) Statistics on litter pup deaths (all litters or a fraction of each litter) from wild-type (WT) or *Tardbp*$^{-/-}$ female mice during first 2 d. (G and H) Pup survival rates from wild-type (WT) or *Tardbp*$^{-/-}$ female mice in first lactation, with six (G) or eight pups (H) per litter. (I) Pup survival rate from *Tardbp*$^{-/-}$ mice nursed by wild-type (WT) mice at L2. (J) Percentage of dead pups with various genotypes. Source data are provided as a Source Data file.

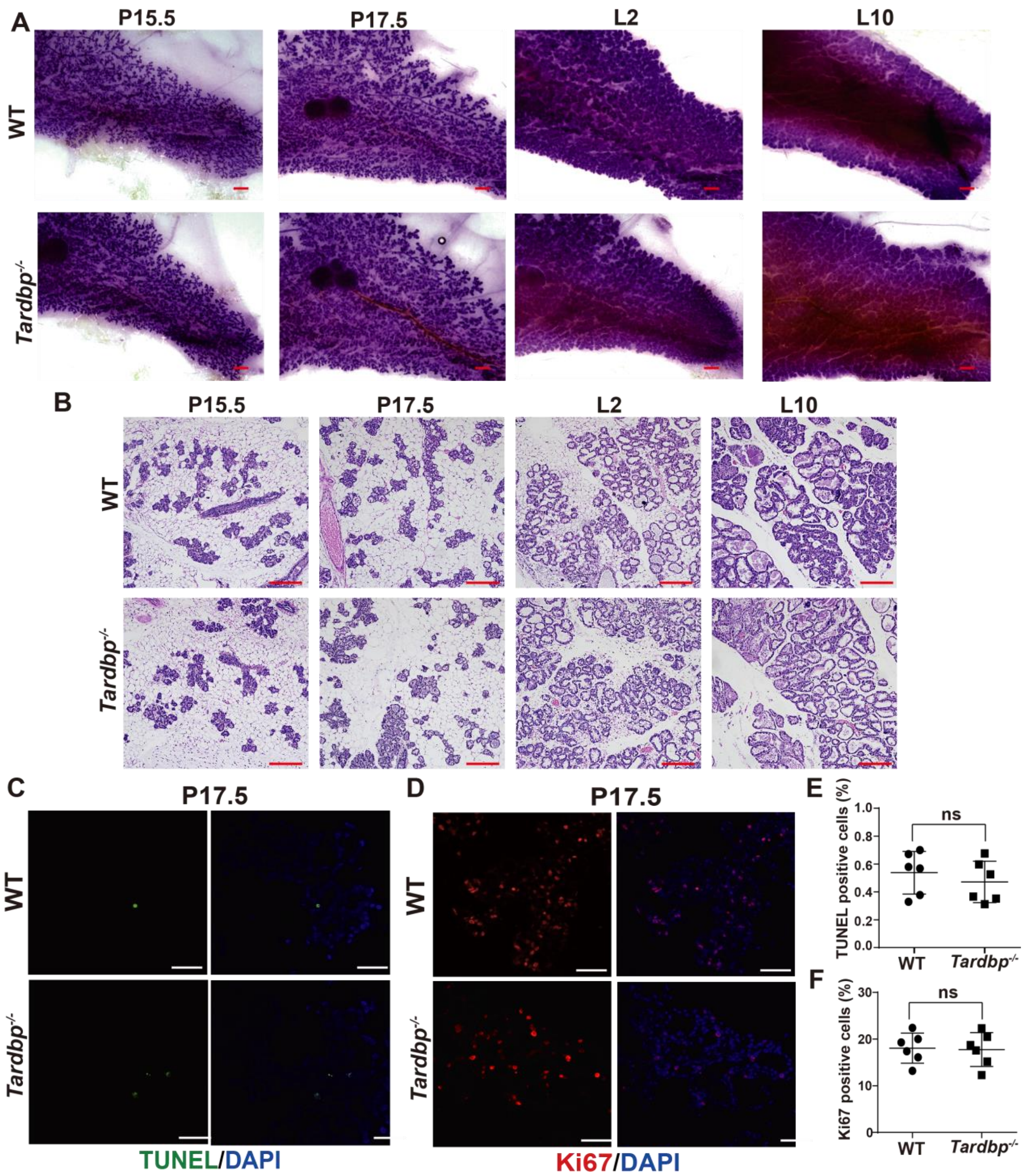

Supplementary Fig. 6. TDP-43 KO displays normal lobuloalveolar development

(A) Whole-mount carmine staining of mammary glands at pregnancy day 15.5 (P15.5), P17.5, lactation day 2 (L2), and L10. Scale bar: 1 mm. (B) Hematoxylin and eosin staining of mammary glands at pregnancy day 15.5 (P15.5), P17.5, lactation day 2 (L2), and L10. Scale bar: 200 μm. (C-F) TUNEL assay (C and E) and Ki67 staining (D and F) showing cell apoptosis and proliferation in mammary glands at P17.5 (n = 6 for each genotype). Scale bar: 50 μm. The results were from n=6 biologically

independent sample. Data are means ± SD. Unpaired *t*-test was used to evaluate statistical significance. ns, not significant. Source data are provided as a Source Data file.

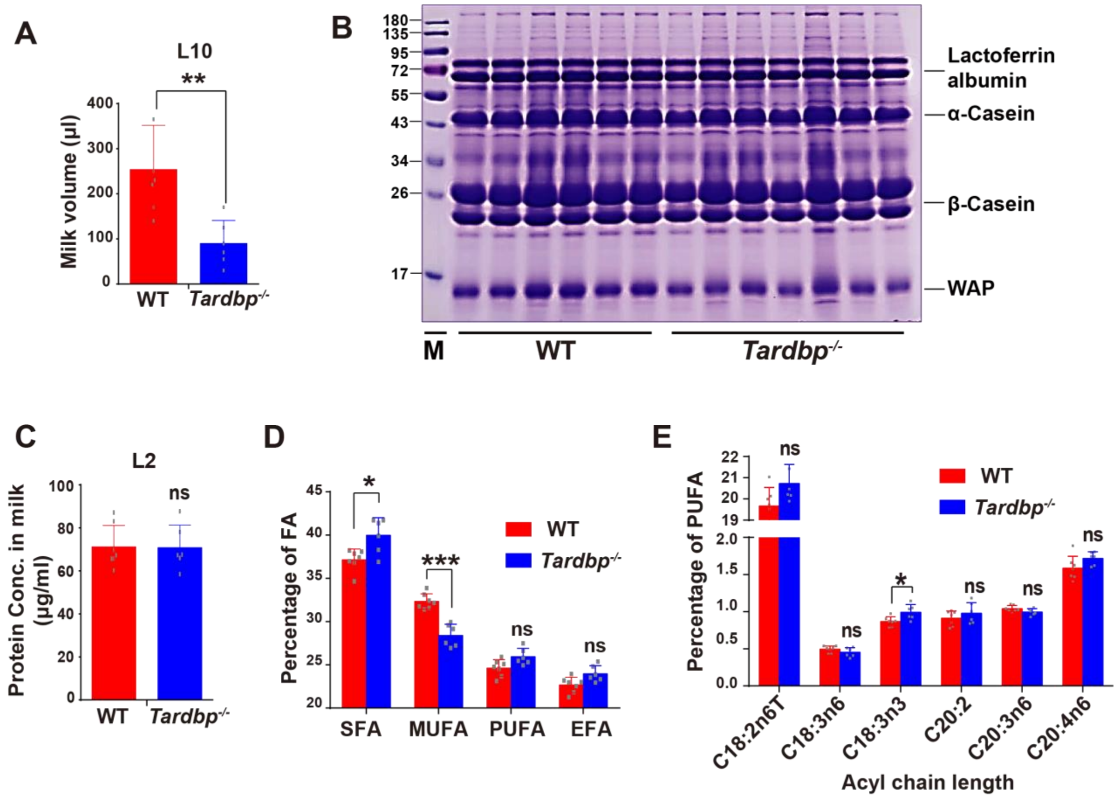

Supplementary Fig. 7. Loss of TDP-43 does not change milk protein composition

(A) Analysis of milk volume from mammary glands of wild-type (WT) and *Tardbp*$^{-/-}$ (KO) female mice following 0.2 units of oxytocin stimulation at lactation day 10 (L10). WT, n = 7 mice; KO, n = 6 mice. (B) Milk proteins at L2 were separated on a 15% SDS-polyacrylamide gel and stained with Coomassie Brilliant Blue. Molecular weight markers are indicated on left. (C) Concentrations of total milk protein analyzed on L2 between indicated genotypes. WT, n = 7 mice; KO, n = 6 mice. (D

and E) Total lipids extracted from milk and quantified by gas-chromatography. FA, fatty acids; SFA, saturated fatty acids; MUFA, monounsaturated fatty acids; PUFA, polyunsaturated fatty; EFA, essential fatty acid. WT, n = 7 mice; KO, n = 6 mice. Data are means ± SD. Unpaired *t*-test was used to evaluate statistical significance. ∗ *P* < 0.05, ∗∗ *P* < 0.01, ∗∗∗ *P* < 0.001. ns, not significant. Source data are provided as a Source Data file.

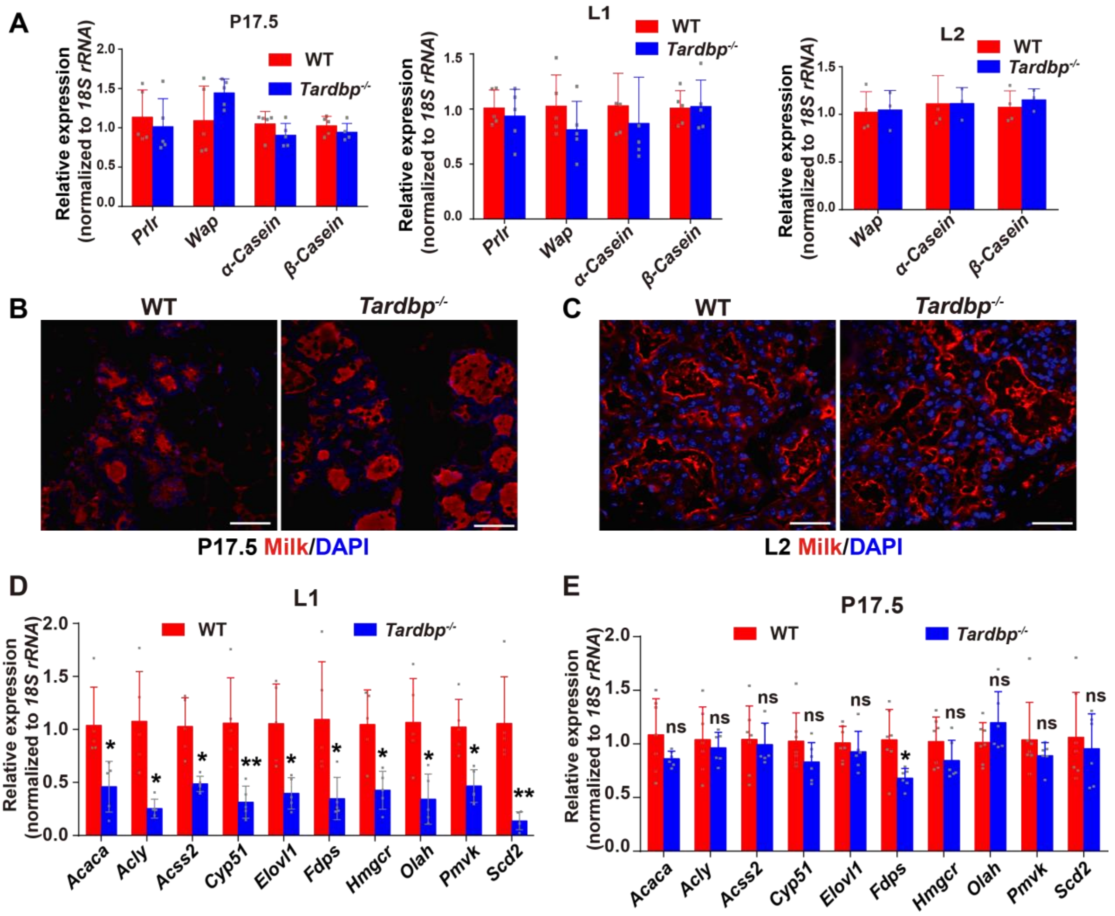

Supplementary Fig. 8. Several milk-related genes are unaffected by *Tardbp* loss

(A) Expression of milk-protein related genes analyzed by qRT-PCR between genotypes at pregnancy day 17.5 (P17.5, left, n = 5 for each genotype), lactation day 1

(L1, middle, n = 5 for each genotype), and L2 (right, WT, n = 4 mice; KO, n = 3 mice). (B and C) Immunofluorescence staining of milk proteins with milk antibodies (red) and DAPI (blue) to stain nuclei at P17.5 (B) and L2 (C). Scale bar: 50 μm. (D and E) Expression of genes involved in lipid synthesis analyzed by qRT-PCR between genotypes at L1 (D) (n = 5 for each genotype) and P17.5 (E) (WT, n = 7 mice; KO, n = 6 mice). Data are means ± SD. Unpaired *t*-test was used to evaluate statistical significance. ∗ $P < 0.05$. ns, not significant. Source data are provided as a Source Data file.

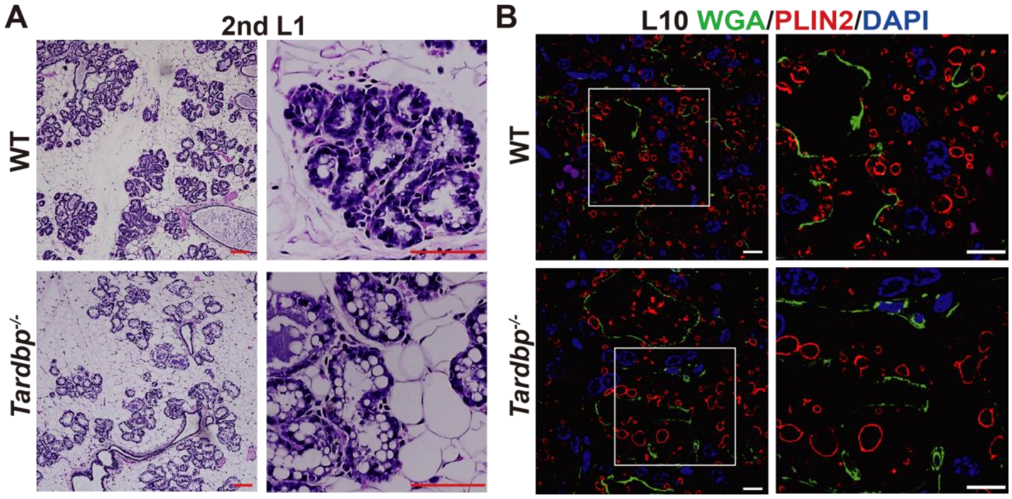

Supplementary Fig. 9. Lipid droplets accumulate in TDP-43 KO alveolar

(A) Hematoxylin and eosin staining of mammary glands at lactation day 1 (L1) in second gestation. Scale bar: 100 μm. (B) Sections of mammary glands collected from both wild-type (WT) and *Tardbp*$^{-/-}$ mice at L10 containing PLIN2 (red), WGA (green), and DAPI (blue). Magnified areas (right) are shown in white boxes (left). Scale bar: 10 μm.

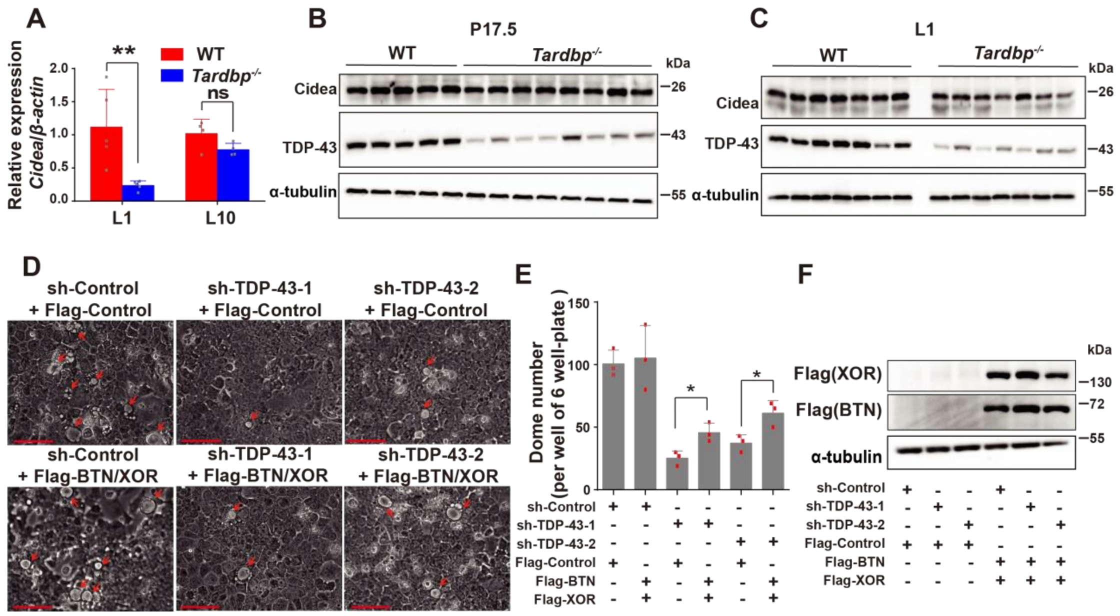

Supplementary Fig. 10. TDP-43 regulates expression of BTN and XOR

(A) Relative mRNA expression of *Cidea* at lactation day 1 (L1) (n = 5 for each genotype)and L10 (n = 4 for each genotype). (B and C) Western blot analysis of Cidea expression at pregnancy day 17.5 (P17.5) (B) and L1 (C). (D and E) Morphology (D) and statistics (E) of dome formation during *in vitro* differentiation of HC11 cells expressed with indicated vectors. Arrowhead indicates structure of dome. Data were shown as the mean ± SD of three independent experiments. Scale bar: 100 μm. (F) Western blot analysis of HC11 cells expressed with indicated vectors. Data are means ± SD. Unpaired *t*-test was used to evaluate statistical significance. $* P < 0.05$. $** P < 0.01$. ns, not significant. Source data are provided as a Source Data file.

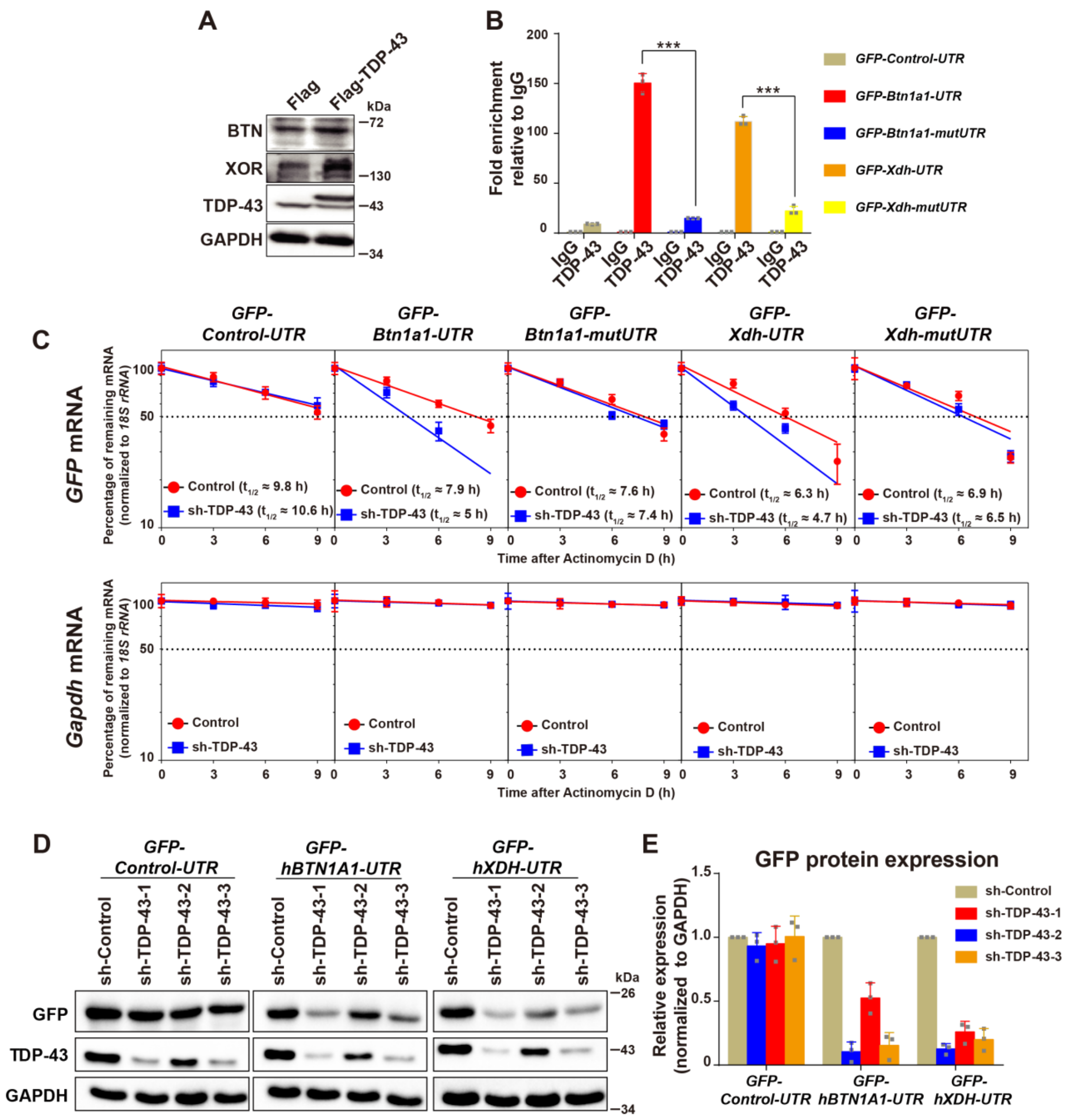

Supplementary Fig. 11. TDP-43 regulates mRNA stability of *Btn1a1* and *Xdh*

(A) Protein levels of BTN and XOR in differentiated HC11 cell line upon TDP-43 overexpression. (B) RNA immunoprecipitation (RIP) assay for analysis of interaction between TDP-43 protein and green fluorescent protein (GFP) reporter mRNA using TDP-43 antibody. (C) *GFP* mRNA stability measured by qPCR 72 h after transfection with GFP reporter and sh-TDP-43. Data were normalized to *18S rRNA* levels in each experiment and represented as a percentage of mRNA levels measured at time 0 h (before actinomycin D addition) using a semi-logarithmic scale. Half-lives ($t_{1/2}$) were

calculated as time of each mRNA to decrease to 50% of its initial abundance. (D) *GFP-hBtn1a1-UTR* and *GFP-hXdh-UTR* were generated by inserting human *Btn1a1* and *Xdh* mRNA following the GFP gene into a mammalian expression vector. GFP protein expression levels were measured by Western blotting 72 h after co-transfection with GFP reporter and sh-TDP-43. (E) Semi-quantitative analyses of the relative levels of GFP protein to sh-Control in each group, which were normalized to GAPDH protein levels. sh-TDP-43-1, sh-TDP-43-2, and sh-TDP-43-3 represent independent shRNAs used. Data were shown as the mean ± SD of three independent experiments. Unpaired *t*-test was used to evaluate statistical significance. *** $P < 0.001$. Source data are provided as a Source Data file.

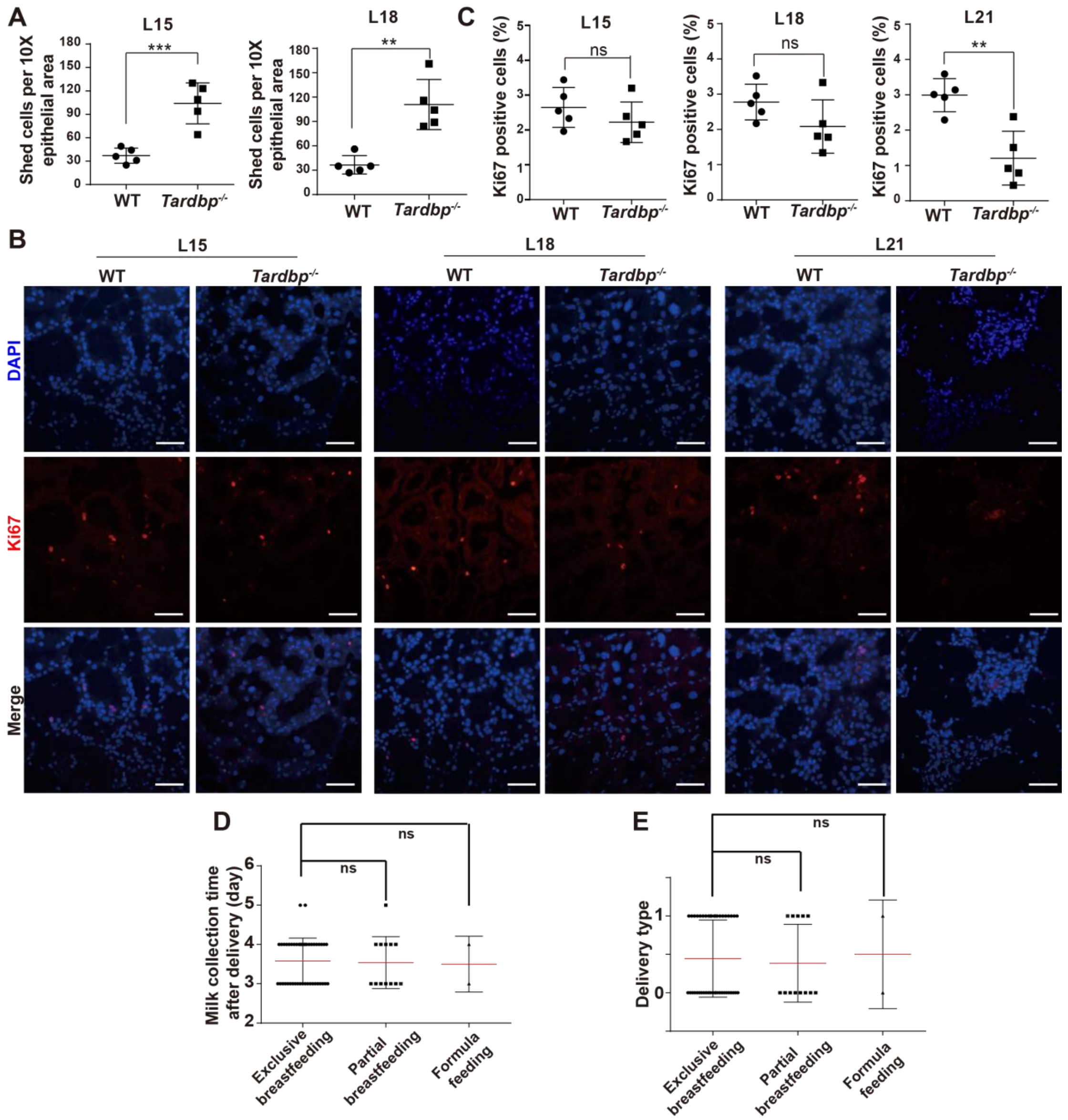

Supplementary Fig. 12. TDP-43 loss results in early involution

(A) Quantification of cells shed into lumen at lactation day 15 (LD15) and LD18 (n = 5 for each genotype). (B-C) Immunostaining and quantification of Ki67 in mammary glands at L15, L18, and L21 (n = 5 for each genotype). Scale bar: 50 μm. (D and E) The milk collection time after delivery (D) and delivery type (E) were analyzed among different groups. Delivery type: 0 represents cesarean delivery, 1 represents spontaneous delivery (E). Exclusive breastfeeding, n = 45; Partial breastfeeding, n = 13; Formula-feeding, n = 2. Data are means ± SD. Unpaired *t*-test was used to

evaluate statistical significance. $**$ $P < 0.01$ $***$ $P < 0.001$. ns, not significant. Source data are provided as a Source Data file.

Supplementary Table 1: Positive selection analysis of 60 one to one orthologous groups of RBPs (RNA-binding proteins).

Gene symbol	Human Ensembl gene ID	Mouse Ensembl gene ID	lnL0(Model A null)	lnL1(Model A alternative)	2ΔlnL	*P* value	FDR *P* val	dN/dS	Positive selection sites (Bayes Empirical Bayes)	Number of orthol-ogous genes
TARDBP	ENSG00000120948	ENSMUSG0000004145	-5443.24438	-5437.2267	#####	5.22E-04	3.13E-02	371.36679	352P0.973*, 360Q0.538	13
SRSF9	ENSG00000111786	ENSMUSG0000002953	-1846.97874	-1852.6746	#####	7.38E-04	2.21E-02	12.40579	1N0.722, 22V0.760, 44R0.734, 129R0.652, 159R0.672	9
G3BP2	ENSG00000138757	ENSMUSG0000002940	-2729.96792	-2726.5703	6.80	9.14E-03	1.83E-01	35.14559		15
TUT1	ENSG00000149016	ENSMUSG0000007164	-6424.90776	-6421.5493	6.72	9.55E-03	1.43E-01	3.45634		11
SFPQ	ENSG00000116560	ENSMUSG0000002882	-4622.4708	-4619.3103	6.32	1.19E-02	1.43E-01	42.88407		14
LIN28A	ENSG00000131914	ENSMUSG0000005096	-1739.10158	-1736.5765	5.05	2.46E-02	2.46E-01	243.74465		14
RBM28	ENSG00000106344	ENSMUSG0000002970	-3940.42809	-3938.2934	4.27	3.88E-02	3.33E-01	88.36565		9
RBM45	ENSG00000155636	ENSMUSG0000004236	-5315.56773	-5313.8716	3.39	6.55E-02	4.91E-01	999.00000		13
ZC3H14	ENSG00000100722	ENSMUSG0000002101	-11894.5651	-11893.502	2.13	1.45E-01	9.66E-01	6.53573		14
SRSF2	ENSG00000161547	ENSMUSG0000003412	-930.269899	-929.36176	1.82	1.78E-01	1.00E+00	10.12855		13
ZCRB1	ENSG00000139168	ENSMUSG0000002263	-2790.6329	-2789.8314	1.60	2.05E-01	1.00E+00	998.99970		13
PPRC1	ENSG00000148840	ENSMUSG0000005549	-4666.16867	-4665.3817	1.57	2.10E-01	1.00E+00	999.00000		14
RBM41	ENSG00000089682	ENSMUSG0000003143	-3155.93072	-3155.5214	0.82	3.66E-01	1.00E+00	3.55707		13
ESRP2	ENSG00000103067	ENSMUSG0000008412	-7808.15568	-7807.7709	0.77	3.80E-01	1.00E+00	80.78809		15
SRSF10	ENSG00000188529	ENSMUSG0000002867	-1057.40708	-1057.316	0.18	6.70E-01	1.00E+00	999.00000		15
SRSF7	ENSG00000115875	ENSMUSG0000002409	-987.527187	-987.47212	0.11	7.40E-01	1.00E+00	1.00000		13
SNRPA	ENSG00000077312	ENSMUSG0000006147	-1222.0103	-1221.9788	0.06	8.02E-01	1.00E+00	140.54418		14
HNRNPA2B1	ENSG00000122566	ENSMUSG0000000498	-3057.54584	-3057.546	0.00	9.87E-01	1.00E+00	5.14002		15
HNRNPL	ENSG00000104824	ENSMUSG0000001516	-1096.65087	-1096.6509	0.00	9.96E-01	1.00E+00	1.00000		13
QKI	ENSG00000112531	ENSMUSG0000006207	-2351.9505	-2351.9505	0.00	9.97E-01	1.00E+00	38.22798		14
PCBP2	ENSG00000197111	NONE	-1384.12485	-1384.1249	0.00	9.97E-01	1.00E+00	33.21347		12
U2AF2	ENSG00000063244	ENSMUSG0000003043	-2682.24999	-2682.25	0.00	9.98E-01	1.00E+00	2.75290		9
MBNL1	ENSG00000152601	ENSMUSG0000002776	-3588.46282	-3588.4628	0.00	9.98E-01	1.00E+00	1.00000		15
RBM3	ENSG00000102317	ENSMUSG0000003116	-926.918433	-926.91843	0.00	1.00E+00	1.00E+00	1.00000		9
RBM8A	ENSG00000265241	ENSMUSG0000003837	-1024.50916	-1024.5092	0.00	1.00E+00	1.00E+00	1.00000		9
HNRNPK	ENSG00000165119	ENSMUSG0000002154	-3442.41546	-3442.4155	0.00	1.00E+00	1.00E+00	1.00000		14
RBMS1	ENSG00000153250	ENSMUSG0000002697	-5030.60193	-5030.6019	0.00	1.00E+00	1.00E+00	1.00000		15
RBM46	ENSG00000151962	ENSMUSG0000003388	-6364.89896	-6364.899	0.00	1.00E+00	1.00E+00	1.00000		15
A1CF	ENSG00000148584	ENSMUSG0000005259	-7089.22834	-7089.2283	0.00	1.00E+00	1.00E+00	1.00000		15
RBMS3	ENSG00000144642	ENSMUSG0000003960	-2164.08665	-2164.0867	0.00	1.00E+00	1.00E+00	1.00000		15
HNRPLL	ENSG00000143889	ENSMUSG0000002409	-1678.2462	-1678.2462	0.00	1.00E+00	1.00E+00	1.00000		15
SRSF1	ENSG00000136450	ENSMUSG0000001837	-292.786263	-292.78626	0.00	1.00E+00	1.00E+00	2.14231		15
IGF2BP3	ENSG00000136231	ENSMUSG0000002981	-2077.68608	-2077.6861	0.00	1.00E+00	1.00E+00	1.00000		14
ZC3H10	ENSG00000135482	ENSMUSG0000003981	-1420.40654	-1420.4065	0.00	1.00E+00	1.00E+00	1.00000		11
KHDRBS3	ENSG00000131773	ENSMUSG0000002233	-2804.70132	-2804.7013	0.00	1.00E+00	1.00E+00	1.00000		15
ANKHD1	ENSG00000131503	ENSMUSG0000002448	-597.184105	-597.18411	0.00	1.00E+00	1.00E+00	1.00000		14
FXR2	ENSG00000129245	ENSMUSG0000001876	-1712.17273	-1712.1727	0.00	1.00E+00	1.00E+00	1.00000		11
HNRNPH2	ENSG00000126945	ENSMUSG0000004542	-3865.15883	-3865.1588	0.00	1.00E+00	1.00E+00	1.00000		12
RALY	ENSG00000125970	ENSMUSG0000002759	-1570.34345	-1570.3435	0.00	1.00E+00	1.00E+00	1.00000		12
ENOX1	ENSG00000120658	ENSMUSG0000002201	-4570.91867	-4570.9187	0.00	1.00E+00	1.00E+00	1.00000		15
TIA1	ENSG00000116001	ENSMUSG0000007133	-1576.56496	-1576.565	0.00	1.00E+00	1.00E+00	1.00000		14
FXR1	ENSG00000114416	ENSMUSG0000002768	-7142.79017	-7142.7902	0.00	1.00E+00	1.00E+00	1.00000		15
CPEB4	ENSG00000113742	ENSMUSG0000002030	-8491.95463	-8491.9546	0.00	1.00E+00	1.00E+00	1.00000		15
RBM24	ENSG00000112183	ENSMUSG0000003813	-774.504356	-774.50436	0.00	1.00E+00	1.00E+00	1.00000		15
FMR1	ENSG00000102081	ENSMUSG0000000083	-5843.18462	-5843.1846	0.00	1.00E+00	1.00E+00	1.00000		15
PABPN1	ENSG00000100836	ENSMUSG0000002219	-1516.87618	-1516.8762	0.00	1.00E+00	1.00E+00	1.00000		10
HNRNPC	ENSG00000092199	ENSMUSG0000006037	-1934.25039	-1934.2504	0.00	1.00E+00	1.00E+00	1.00000		10
PABPC4	ENSG00000090621	ENSMUSG0000001125	-5639.90163	-5639.9016	0.00	1.00E+00	1.00E+00	1.00000		14
FUS	ENSG00000089280	ENSMUSG0000003079	-2554.52859	-2554.5286	0.00	1.00E+00	1.00E+00	1.00000		12
CNOT4	ENSG00000080802	ENSMUSG0000003878	-7403.92991	-7403.9299	0.00	1.00E+00	1.00E+00	1.00000		15
SART3	ENSG00000075856	ENSMUSG0000001897	-5739.92708	-5739.9271	0.00	1.00E+00	1.00E+00	1.00000		14
ZNF638	ENSG00000075292	ENSMUSG0000003001	-9515.92421	-9515.9242	0.00	1.00E+00	1.00E+00	1.00000		11

IGF2BP2	ENSG00000073792	ENSMUSG0000003358	-4202.3226	-4202.3226	0.00	1.00E+00	1.00E+00	1.00000		14
DAZAP1	ENSG00000071626	ENSMUSG0000006956	-1710.87971	-1710.8797	0.00	1.00E+00	1.00E+00	1.00000		13
PABPC1	ENSG00000070756	ENSMUSG0000002228	-6197.34954	-6197.3495	0.00	1.00E+00	1.00E+00	8.85527		14
HuR	ENSG00000066044	ENSMUSG0000004002	-1360.50661	-1360.5066	0.00	1.00E+00	1.00E+00	2.10399		14
YBX1	ENSG00000065978	NONE	-1798.74983	-1798.7498	0.00	1.00E+00	1.00E+00	1.00000		13
SAMD4A	ENSG00000020577	ENSMUSG0000002183	-1380.96807	-1380.9681	0.00	1.00E+00	1.00E+00	1.00000		14
MATR3	ENSG00000015479	ENSMUSG0000003723	-9662.01709	-9662.0171	0.00	1.00E+00	1.00E+00	1.00000		15
RBM5	ENSG00000003756	ENSMUSG0000003258	-9497.61194	-9497.6119	0.00	1.00E+00	1.00E+00	1.00000		15

Supplementary Table 2: Analysis of UG motif for lipid-related genes upon *Tardbp* KO.

Gene_ Symbol	Ensembl_Gene_ID	Transcript_ID	Transcript_Length	Numbmer_of_UG_Island	Numbmer_of_UG_element	*P*_value
Btn1a1	ENSMUSG00000000706	ENSMUST00000041674	3423	6	39	1.49349E-17
Cptp	ENSMUSG00000029073	ENSMUST00000030950	2662	6	18	0.000357381
Prkaa2	ENSMUSG00000028518	ENSMUST00000030243	7969	2	6	0.007117038
Pafah2	ENSMUSG00000037366	ENSMUST00000105870	3180	2	6	0.007289426
Ldlr	ENSMUSG00000032193	ENSMUST00000034713	4627	2	6	0.007305817
Ldlr	ENSMUSG00000032193	ENSMUST00000034713	4627	2	6	0.007305817
Thrsp	ENSMUSG00000035686	ENSMUST00000043077	1277	2	6	0.008034018
Serinc5	ENSMUSG00000021703	ENSMUST00000049488	5366	5	15	0.0088084
Acaca	ENSMUSG00000020532	ENSMUST00000103201	9513	5	15	0.010939907
Scd2	ENSMUSG00000025203	ENSMUST00000026221	5455	1	3	0.021846701
Elovl1	ENSMUSG00000006390	ENSMUST00000006557	1811	1	6	0.022561794
Acat1	ENSMUSG00000032047	ENSMUST00000034547	3405	1	3	0.023867566
Cidea	ENSMUSG00000024526	ENSMUST00000025404	1115	1	3	0.028664448
Ano4	ENSMUSG00000035189	ENSMUST00000182462	4117	2	6	0.0394009
Agpat1	ENSMUSG00000034254	ENSMUST00000037489	2190	4	12	0.046288387
Degs2	ENSMUSG00000021263	ENSMUST00000167978	1729	3	9	0.051332396
Acacb	ENSMUSG00000042010	ENSMUST00000102582	8788	4	12	0.081294368
Mfsd2a	ENSMUSG00000028655	ENSMUST00000030408	2151	1	3	0.136583128
Lss	ENSMUSG00000033105	ENSMUST00000048678	4667	4	12	0.13830437
Pcyt2	ENSMUSG00000025137	ENSMUST00000026129	1881	1	3	0.176644528
Akt1	ENSMUSG00000001729	ENSMUST00000001780	2690	2	6	0.179445526
Acly	ENSMUSG00000020917	ENSMUST00000107389	4426	0	0	0.180791181
Lpcat1	ENSMUSG00000021608	ENSMUST00000022099	3675	3	9	0.191077933
Stard10	ENSMUSG00000030688	ENSMUST00000163799	1723	2	6	0.194491013
Steap2	ENSMUSG00000015653	ENSMUST00000115426	10408	5	15	0.259507856
Osbpl1a	ENSMUSG00000044252	ENSMUST00000074352	3956	4	12	0.276220722
Ptk2b	ENSMUSG00000059456	ENSMUST00000022622	4034	4	12	0.281429224
Gdpd1	ENSMUSG00000061666	ENSMUST00000020804	2336	2	6	0.286103013
Insig1	ENSMUSG00000045294	ENSMUST00000059155	2697	1	3	0.383367431
Fads1	ENSMUSG00000010663	ENSMUST00000010807	3460	2	6	0.384268267
Cpt1a	ENSMUSG00000024900	ENSMUST00000025835	4317	1	3	0.495490318
Msmo1	ENSMUSG00000031604	ENSMUST00000034015	2044	2	6	0.495805639
Apol7c	ENSMUSG00000044309	ENSMUST00000062562	2060	1	3	0.501569428
Plcl2	ENSMUSG00000038910	ENSMUST00000043938	4185	3	9	0.513307245
Cyp1b1	ENSMUSG00000024087	ENSMUST00000024894	5128	3	9	0.590404216
Olah	ENSMUSG00000026645	ENSMUST00000194918	1764	1	3	0.626506996
Pcx	ENSMUSG00000024892	ENSMUST00000068004	4148	1	3	0.628844814
Smpdl3b	ENSMUSG00000028885	ENSMUST00000030709	2006	0	0	0.637352221
Smpdl3b	ENSMUSG00000028885	ENSMUST00000030709	2006	0	0	0.637352221
Oxct1	ENSMUSG00000022186	ENSMUST00000110690	3527	2	6	0.804679378
Mvd	ENSMUSG00000006517	ENSMUST00000211883	1833	1	3	0.805951894
Acap1	ENSMUSG00000001588	ENSMUST00000001631	2482	0	0	1
Fdps	ENSMUSG00000059743	ENSMUST00000196709	1382	0	0	1
Aacs	ENSMUSG00000029482	ENSMUST00000031445	3239	2	6	1

Supplementary Table 3. Primers and shRNA sequences used in this paper

**Primers**	**Sequence**
sh-mus-TDP-43-1F	CCGGGGAGAGGATTTGATCATTAAACTCGAGTTTAATGATCAAAT CCTCTCCTTTTTG
sh-mus-TDP-43-1R	AATTCAAAAAGGAGAGGATTTGATCATTAAACTCGAGTTTAATGA TCAAATCCTCTCC
sh-mus-TDP43-2F	CCGGCTTTGTTCGATTTACAGAATACTCGAGTATTCTGTAAATC GAACAAAGTTTTTG
sh-mus-TDP43-2R	AATTCAAAAACTTTGTTCGATTTACAGAATACTCGAGTATTCTG TAAATCGAACAAAG
sh-mus-TDP43-3F	CCGGGCAAAGATGTCTGAATATATTCTCGAGAATATATTCAGAC ATCTTTGCTTTTTG
sh-mus-TDP43-3R	AATTCAAAAAGCAAAGATGTCTGAATATATTCTCGAGAATATAT TCAGACATCTTTGC
Sh-mus-SRSF9-1F	CCGGTGGAAGAAACGGTTACGATTACTCGAGTAATCGTAACCGT TTCTTCCATTTTTG
Sh-mus-SRSF9-1R	AATTCAAAAATGGAAGAAACGGTTACGATTACTCGAGTAATCG TAACCGTTTCTTCCA
Sh-mus-SRSF9-2R	CCGGCGTAAACTGGATGACACCAAACTCGAGTTTGGTGTCATCC AGTTTACGTTTTTG
Sh-mus-SRSF9-2R	AATTCAAAAACGTAAACTGGATGACACCAAACTCGAGTTTGGT GTCATCCAGTTTACG
Xdh-F	ATGACGAGGACAACGGTAGAT
Xdh-R	TCATACTTGGAGATCATCACGGT
Btn1a1-F	ACGTCAGAGTCCAAGAAGCAT
Btn1a1-R	AGGCCAGTAAGATGATAGCCA
mus-Tdp43-1F	GAGTGCCTCTGTGCATTTGA
mus-Tdp43-1R	TTCCCATTGATATGCTCTGCT
mus-Tdp43-2F	GACCACTCAGAAGTGTGTGGG
mus-Tdp43-2R	TCCTCAAATTCGACGAAAGCAAA
Cidea-F	TGACATTCATGGGATTGCAGAC
Cidea-R	GGCCAGTTGTGATGACTAAGAC
Acly-F	ACCCTTTCACTGGGGATCACA
Acly-R	GACAGGGATCAGGATTTCCTTG
Acat1-F	CAGGAAGTAAGATGCCTGGAAC
Acat1-R	TTCACCCCCTTGGATGACATT
Acss1-F	GTTTGGGACACTCCTTACCATAC
Acss1-R	AGGCAGTTGACAGACACATTC
Acss2-F	AAACACGCTCAGGGAAAATCA
Acss2-R	ACCGTAGATGTATCCCCCAGG
Fasn-F	GGAGGTGGTGATAGCCGGTAT
Fasn-R	TGGGTAATCCATAGAGCCCAG
Acaca-F	GATGAACCATCTCCGTTGGC
Acaca-R	GACCCAATTATGAATCGGGAGTG

Acacb-F	CGCTCACCAACAGTAAGGTGG
Acacb-R	GCTTGGCAGGGAGTTCCTC
Elovl1-F	TCCAAAGCTACCCTCTGATGG
Elovl1-R	AGGGAGAGTATCACCAGTGAGA
Elovl5-F	ATGGAACATTTCGATGCGTCA
Elovl5-R	GTCCCAGCCATACAATGAGTAAG
Fads1-F	AGCACATGCCATACAACCATC
Fads1-R	TTTCCGCTGAACCACAAAATAGA
Msmo1-F	AAACAAAAGTGTTGGCGTGTTC
Msmo1-R	AAGCATTCTTAAAGGGCTCCTG
Olah-F	GTTTGTGTCAAAAGCCGGATG
Olah-R	GTTTCTCTCCCAGCCAGTCTTA
Prkaa2-F	CAGGCCATAAAGTGGCAGTTA
Prkaa2-R	AAAAGTCTGTCGGAGTGCTGA
Scd2-F	GCATTTGGGAGCCTTGTACG
Scd2-R	AGCCGTGCCTTGTATGTTCTG
Fads2-F	AAGGGAGGTAACCAGGGAGAG
Fads2-R	CCGCTGGGACCATTTGGTAA
Fdps-F	GGAGGTCCTAGAGTACAATGCC
Fdps-R	AAGCCTGGAGCAGTTCTACAC
Lss-F	TCGTGGGGGACCCTATAAAAC
Lss-R	CGTCCTCCGCTTGATAATAAGTC
Mvd-F	ATGGCCTCAGAAAAGCCTCAG
Mvd-R	TGGTCGTTTTTAGCTGGTCCT
Pmvk-F	AAAATCCGGGAAGGACTTCGT
Pmvk-R	AGAGCACAGATGTTACCTCCA
Cyp51-F	GACAGGAGGCAACTTGCTTTC
Cyp51-R	GTGGACTTTTCGCTCCAGC
Hmgcr-F	AGCTTGCCCGAATTGTATGTG
Hmgcr-R	TCTGTTGTGAACCATGTGACTTC
Nsdhl-F	TCATGGTGAATCAAAGCGAGG
Nsdhl-R	CCGGGGGTTATCAAAGCCTTG
β-actin-F	GGCTGTATTCCCCTCCATCG
β-actin-R	CCAGTTGGTAACAATGCCATGT
Rps18-F	TTCGGAACTGAGGCCATGAT
Rps18-R	TTTCGCTCTGGTCCGTCTTG
Gapdh-F	AGGTCGGTGTGAACGGATTTG
Gapdh-R	TGTAGACCATGTAGTTGAGGTCA
α-Casein-F	CAGCATAGCAGCAGTGAGGA
α-Casein-R	CTTCCTGAGCACTTGCCATT
Wap-F	TATCATCTGCCAAACCAACG
Wap-R	GGTCGCTGGAGCATTCTATC
β-Casein-F	GGTGAATCTCATGGGACAGC
β-Casein-R	CACAGGGGGTTGAGCAATAG

Prlr-F	CACTTGCTTACATGCTGCTTG
Prlr-R	CAGGTGGTGACTGTCCATTCA
pcdna-btn-utr-1f	CCAAGCTGGCTAGTTAAGCTTgcaagagacagaaaggag
pcdna-btn-utr-1r	CCACACTGGACTAGTGGATCCacaatagaagtggaagag
homo-cd3e-1f	CCTCTTATCAGTTGGCGTTTGG
homo-cd3e-1r	TTCAGTGACAGGTGATCCTCA
homo-cd68-1f	GGAAATGCCACGGTTCATCCA
homo-cd68-1r	TGGGGTTCAGTACAGAGATGC
homo-gpr97-1f	CGAAGGGCCAAGAAACACCT
homo-gpr97-1r	CGTAGTTTAGCCAGTATCTCTGC
homo-cd18-1f	TGCGTCCTCTCTCAGGAGTG
homo-cd18-1r	GGTCCATGATGTCGTCAGCC
homo-CSN2-1f	CCCTGTGGTCTGTTCCTCAG
homo-CSN2-1r	TGGGTGGGGTTAAGTAGAAGTTC
homo-CSN1S1-1f	CATGCCCAGGAGCAAATTCG
homo-CSN1S1-1r	TTGGAGATGTCGGAAAACGGT
homo-LALBA-1f	GGCATCGCTTTGCCTGAATTG
homo-LALBA-1r	TTGGCACACATTATGTCATCAGT
homo-CSN3-1f	CCAATTTGTACCAACGTAGACCA
homo-CSN3-1r	GGGCATGTGGCCTAACTACAG
homo-hnRNPA1-1f	TCAGAGTCTCCTAAAGAGCCC
homo-hnRNPA1-1r	ACCTTGTGTGGCCTTGCAT
U1snRNA-F	GGGAGATACCATGATCACGAAGGT
U1snRNA-R	CCACAAATTATGCAGTCGAGTTTCCC
18S rRNA-F	GCGGCGGAAAATAGCCTTTG
18S rRNA-R	GATCACACGTTCCACCTCATC
GFP-RIP-F	CGACAACCACTACCTGAGCA
GFP-RIP-R	GAACTCCAGCAGGACCATGT
