# Supplementary material for: TDP-43 facilitates milk lipid secretion by post-transcriptional regulation of Btn1a1 and Xdh
Source: Nat Commun. 2020 Jan 17;11:341. doi: 10.1038/s41467-019-14183-1 (PMC6969145; doi:10.1038/s41467-019-14183-1)
Supplement: Supplementary file 3 — Reporting Summary [file 41467_2019_14183_MOESM3_ESM.pdf]

nature research

Corresponding author(s): Baowei Jiao

Last updated by author(s): Nov 27, 2019

Reporting Summary

Nature Research wishes to improve the reproducibility of the work that we publish. This form provides structure for consistency and transparency in reporting. For further information on Nature Research policies, see Authors & Referees and the Editorial Policy Checklist.

Statistics

For all statistical analyses, confirm that the following items are present in the figure legend, table legend, main text, or Methods section.

n/a	Confirmed	
☐	☒	The exact sample size (*n*) for each experimental group/condition, given as a discrete number and unit of measurement
☐	☒	A statement on whether measurements were taken from distinct samples or whether the same sample was measured repeatedly
☐	☒	The statistical test(s) used AND whether they are one- or two-sided<br>*Only common tests should be described solely by name; describe more complex techniques in the Methods section.*
☒	☐	A description of all covariates tested
☒	☐	A description of any assumptions or corrections, such as tests of normality and adjustment for multiple comparisons
☐	☒	A full description of the statistical parameters including central tendency (e.g. means) or other basic estimates (e.g. regression coefficient) AND variation (e.g. standard deviation) or associated estimates of uncertainty (e.g. confidence intervals)
☒	☐	For null hypothesis testing, the test statistic (e.g. *F*, *t*, *r*) with confidence intervals, effect sizes, degrees of freedom and *P* value noted<br>*Give P values as exact values whenever suitable.*
☒	☐	For Bayesian analysis, information on the choice of priors and Markov chain Monte Carlo settings
☒	☐	For hierarchical and complex designs, identification of the appropriate level for tests and full reporting of outcomes
☒	☐	Estimates of effect sizes (e.g. Cohen's *d*, Pearson's *r*), indicating how they were calculated

Our web collection on statistics for biologists contains articles on many of the points above.

Software and code

Policy information about availability of computer code

Data collection	Microscopy images were taken using NIS-Elements F 4.0. qRT-PCR data were collected using QuantStudio 3. Western blot images were taken using SageCapture software. Electron micrographs were captured by JEM 1400 Plus.The RNA-Seq data were deposited in the NCBI GEO database under ID code: GSE116456.
Data analysis	Graphs and statistical analyses were performed using GraphPad Prism 6. STAR software STAR_2.4.2a (Dobin A et al., 2013), FeatureCounts v1.4.6-p5 (Liao Y et al., 2014) and edgeR package 1.6 (Robinson MD et al., 2014) were used for RNAseq analysis which is briefly described in the manuscript (Methods). Microscopy images were analyzed using Image-Pro Plus 5.

For manuscripts utilizing custom algorithms or software that are central to the research but not yet described in published literature, software must be made available to editors/reviewers. We strongly encourage code deposition in a community repository (e.g. GitHub). See the Nature Research guidelines for submitting code & software for further information.

Data

Policy information about availability of data

All manuscripts must include a data availability statement. This statement should provide the following information, where applicable:

- Accession codes, unique identifiers, or web links for publicly available datasets
- A list of figures that have associated raw data
- A description of any restrictions on data availability

All data in this study are available from the corresponding author upon reasonable request. RNAseq data has been deposited in the GEO under GSE116456 and will be made upon acceptance of the manuscript.

Field-specific reporting

Please select the one below that is the best fit for your research. If you are not sure, read the appropriate sections before making your selection.

☒ Life sciences ☐ Behavioural & social sciences ☐ Ecological, evolutionary & environmental sciences

For a reference copy of the document with all sections, see nature.com/documents/nr-reporting-summary-flat.pdf

Life sciences study design

All studies must disclose on these points even when the disclosure is negative.

Sample size	The sample size in each experiment was determined to give an appropriate power of the test.<br>The sample sizes of different experiments, including statistics of pups weight and immunofluorescence microscopy, were chosen accord ing to the previous studies published by other laboratories.
Data exclusions	No data were excluded.
Replication	All attempts at replication were successful as determined using a statistical analysis.<br>A minimum of 5 individuals (biological replicate) have been processed to perform animal experiments in a minimum of 2 independent experiments (technical replicate) in order to reach statistical significance.<br>All experiments were reliably reproduced.
Randomization	Sample were allocated randomly to experimental groups, except when purpose of the experiment compare difference between control and Tardbp KO mice.
Blinding	All experiments were conducted in a double blinded fashion in which the researchers were blinded to group allocation.<br>Blinded experiments were performed for genes expression analyses of human milk.

Reporting for specific materials, systems and methods

We require information from authors about some types of materials, experimental systems and methods used in many studies. Here, indicate whether each material, system or method listed is relevant to your study. If you are not sure if a list item applies to your research, read the appropriate section before selecting a response.

Materials & experimental systems

n/a	Involved in the study	
☐	☒	Antibodies
☐	☒	Eukaryotic cell lines
☒	☐	Palaeontology
☐	☒	Animals and other organisms
☐	☒	Human research participants
☒	☐	Clinical data

Methods

n/a	Involved in the study	
☒	☐	ChIP-seq
☒	☐	Flow cytometry
☒	☐	MRI-based neuroimaging

Antibodies

Antibodies used	For immunofluorescence staining, antibodies were used as follows: TDP-43 (clone ERP5810, Abcam, ab109535), K14 (clone LL002, Abcam, ab7800), K18 (clone C-04, Abcam, ab668), PLIN2 (Progen, GP40), milk antibody (Nordic Immunology, 5941), Ki67 (Abcam, ab15580), and WGA (Life, 11261). The secondary antibodies used in immunostaining were fluorescein-labeled anti-rabbit (KPL, 02-15-06), fluorescein-labeled anti-mouse (KPL, 02-18-06), cy3 goat anti-mouse (Life, A10521), cy3 goat anti-rabbit (Life, A10520), and TRITC rabbit anti-guinea pig (Life,A1888).<br><br>The antibodies used for immunoblotting were: Flag (CST, #14793), α-tubulin (clone B-5-1-2,Sigma, T5168), GAPDH (Santa Cruz, sc-25778), TDP-43 (clone ERP5810,Abcam, ab109535), XOR (clone EPR4605,Abcam, ab109235), Cidea (Abcam ,ab8402) and BTN (Acris, AP09532SU-N). The secondary antibodies were incubated with horseradish peroxidase (HRP)-linked secondary antibodies: Anti-mouse IgG(Sigma, A4461),Anti-Rabbit(Sigma, A6154),Anti-Guinea Pig(Life, 61-4620).<br><br>For RNA immunoprecipitation, antibodies were used as follows:TDP-43 (Proteintech,10782-2-AP), Flag (CST, #14793), or IgG (Millipore, PP64B).
Validation	Each antibody has been validated by the companies and by the result of the paper.

Eukaryotic cell lines

Policy information about cell lines

Cell line source(s)	HC11 cell line was from Bernd Groner lab, Ludwig Institute for Cancer Research.<br>HC11 cell line was a gift from , Northwest A&F University.
Authentication	None of the cell line used have been authenticated.
Mycoplasma contamination	All the cell lines are mycoplasma-free.
Commonly misidentified lines<br>(See ICLAC register)	No commonly misidentified cell lines were used in the study.

Animals and other organisms

Policy information about studies involving animals; ARRIVE guidelines recommended for reporting animal research

Laboratory animals	C57BL/6（Female, WAP-Cre transgenic mice were purchased from Jackson Laboratory (Stock No. 000664). The Tardbp floxed mice were obtained as a gift from Che-Kun James Shen, Academia Sinica. Tardbp-/-;WAP-Cre mice were generated by crossing Tardbp floxed mice with WAP-Cre mice mice.
Wild animals	The study did not involve wild animals.
Field-collected samples	The study did not involve sample collected from the field.
Ethics oversight	All animal studies comply with relevant ethical regulations for animal testing and research, and were approved by the Institutional Animal Care. Animals were maintained and studies were carried out in accordance with institutional guidelines.

Note that full information on the approval of the study protocol must also be provided in the manuscript.

Human research participants

Policy information about studies involving human research participants

Population characteristics	Population are 60 healthy women who gave birth to term infants.
Recruitment	Fresh human milk were obtained from 60 healthy women on days 3-5 postpartum who gave birth to term infants, which have similar time span of mother pregnancy, times of gestation and pregnancy.
Ethics oversight	The relevant hospital Ethics Committee approved the project. And the informed consent was obtained from all participants.

Note that full information on the approval of the study protocol must also be provided in the manuscript.
